# Robust generation of clinically applicable human pluripotent stem cells from peripheral blood by chemical reprogramming

**DOI:** 10.1038/s41421-025-00852-7

**Published:** 2025-12-23

**Authors:** Xiaodi Fu, Fangqi Peng, Ruyi Cai, Jingping Mao, Tianxing Liu, Yingshuai Dong, Ruoqi Cheng, Zhihan Yang, Guanxian Chen, Cheng Li, Rong Mu, Lin Cheng, Yanglu Wang, Jingyang Guan, Hongkui Deng

**Affiliations:** 1 MOE Key Laboratory of Cell Proliferation and Differentiation, School of Life Sciences and MOE Engineering Research Center of Regenerative Medicine, School of Basic Medical Sciences, State Key Laboratory of Natural and Biomimetic Drugs, Peking University Health Science Center, Peking-Tsinghua Center for Life Sciences, Peking University, Beijing, China; 2 Department of Rheumatology and Immunology, Peking University Third Hospital, Beijing, China; 3 State Key Laboratory of Natural and Biomimetic Drugs, Department of Molecular and Cellular Pharmacology, School of Pharmaceutical Sciences, Peking University, Beijing, China; 4 School of Life Sciences, Center for Bioinformatics, Center for Statistical Science, Peking University, Beijing, China; 5 Changping Laboratory, Beijing, China

**Keywords:** Reprogramming, Induced pluripotent stem cells

Dear Editor,

The derivation of induced pluripotent stem (iPS) cells, which can self-renew and generate unlimited patient-specific functional cells, has revolutionized regenerative medicine^1^. iPS cell-based cell therapies are now achieving promising progress in clinical trials for a range of major diseases^2^. However, the conventional transcription factor-based reprogramming approaches for generating clinical-grade, patient-specific iPS cell lines remain prohibitively expensive, time-consuming, and reliant on complex exogenous gene delivery systems^1–3^, which severely impedes their broader clinical translation.

Small molecule-based chemical reprogramming offers an innovative strategy for generating iPS cells from somatic cells^4^. In 2013, our group pioneered a chemical reprogramming system to convert mouse somatic cells into pluripotent stem cells^5,6^. This method enables highly controllable, flexible and simple manipulation of cell fates, through precise targeting of signaling and epigenetic pathways^4^. In 2022, we successfully employed this approach to generate human chemically induced pluripotent stem (hCiPS) cells from human somatic cells^7^, and further illustrate the stepwise nature of chemical reprogramming to highlight its fundamental differences from transcription factor-based reprogramming strategies^7^. Notably, small molecules are readily synthesized, standardized, cost-effective and non-integrative^4^, providing distinct advantages for manufacturing of stem cells for clinical translations. Importantly, our subsequent clinical results demonstrated that transplantation of autologous hCiPS cell-derived islets into a type 1 diabetes patient led to sustained insulin independence^8^, underscoring the therapeutic potential of patient-specific hCiPS cells for treating major diseases. These advances position chemical reprogramming as a highly promising strategy for stem cell production and a powerful platform for personalized cell-based clinical applications.

For scalable clinical translation, reprogramming human peripheral blood cells into hCiPS cells represents a highly promising pathway, owing to the unparalleled accessibility, convenience, and abundance of blood cells. Additionally, due to the simplicity and minimal invasiveness of blood collection, coupled with the wide availability of banked samples, blood-based reprogramming could provide a practical and scalable strategy for generating patient-specific hCiPS cell lines. Importantly, major limitations remain with current transcription factor-based reprogramming approaches for blood cells^9^. For instance, Sendai virus and episomal-based techniques are still low efficient and require extended culture periods to eliminate residual reprogramming agents, complicating their clinical applications^9^. Therefore, developing a robust, reproducible, cost-effective, convenient and clinically compliant reprogramming system for human blood cells is essential to greatly accelerate stem cell-based therapeutic translation.

To establish a clinical applicable blood reprogramming system, we first attempted to directly adapt our recently established serum-based reprogramming protocol^10^ by eliminating fetal bovine serum (FBS), as these animal-derived components introduce potential risks of pathogen contaminations and increase immunogenicity, thus not suitable for further clinical translation. However, the erythroid progenitor cells (EPCs) derived from peripheral blood mononuclear cells (PBMCs) failed to respond to this serum-removed condition (Fig. 1a). We did not observe the emergence of epithelial-like cell clusters that typically marks the initial phase of successful reprogramming (Fig. 1a). This complete absence of morphological transformation, coupled with the lack of proliferative response in the cultured cells (Fig. 1b), indicated that serum played an essential role in facilitating the early cell fate transition.

**Fig. 1:**
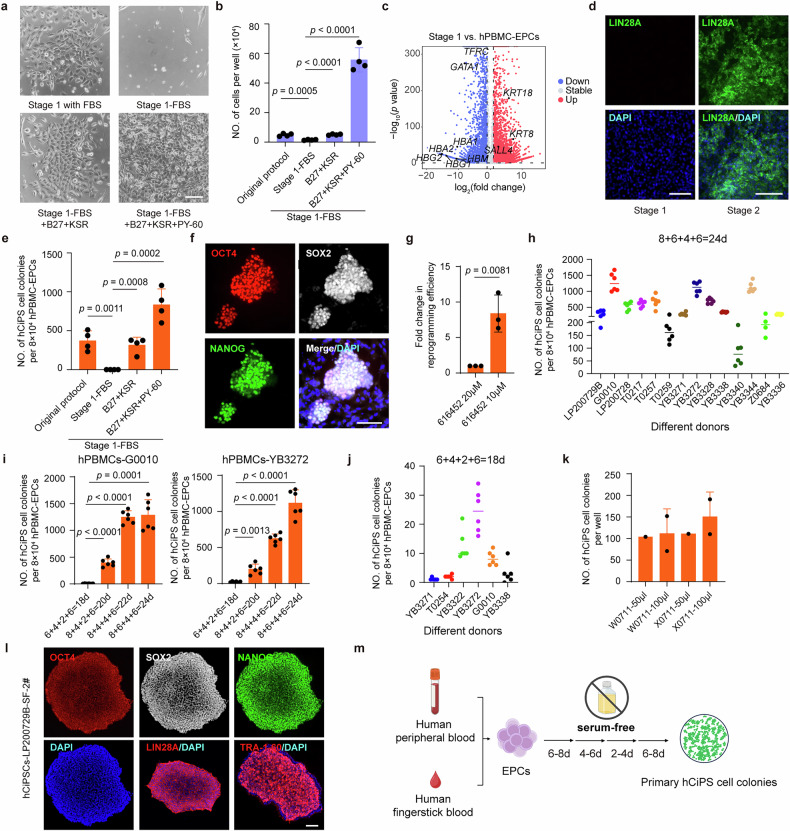
Establishment of a clinically-compliant reprogramming protocol for blood cells. **a** Representative brightfield images of cell morphologies under the indicated conditions. EPCs were cultured using the original stage 1 protocol with FBS, stage 1–FBS, stage 1–FBS + B27 + KSR, or stage 1–FBS + B27 + KSR + PY-60 conditions. Scale bar, 100 μm. **b** Quantification of cell yields across the indicated conditions. The numbers of cells harvested from cultures using the original protocol with the indicated conditions were counted (*n* = 4). Error bars indicate standard deviation (SD) of 4 biological replicates. **c** Volcano plot of gene expression changes between stage 1 and hPBMC-EPCs. Red dots represent upregulated genes, and blue dots represent downregulated genes. **d** Immunofluorescence staining of LIN28A (green) in cells at the end of stage 1 and stage 2. Scale bars, 100 μm. Data are representative of three independent experiments. **e** Numbers of hCiPS cell colonies at the end of chemical reprogramming with different stage 1 culture conditions (*n* = 4). Error bars indicate SD of 4 biological replicates. **f** Immunofluorescent staining of pluripotency markers in the primary hCiPS cell colonies. Scale bar, 100 μm. **g** Fold change in reprogramming efficiency under different concentrations of 616452 (20 μM and 10 μM) in stage 1 (*n* = 4). The baseline refers to the condition treated with 20 µM of 616452. Error bars indicate SD of 4 biological replicates. **h** Numbers of hCiPS cell colonies from different donors (for cell line Z0684, *n* = 4; for other samples, *n* = 6). “8 + 4 + 4 + 6” represents a sequential duration (in days) for each stage. The short line indicates the mean of the indicated biological replicates. **i** Reprogramming kinetics for hPBMCs-G0010 and hPBMCs-YB3272 under different durations of stages (*n* = 6). Error bars indicate SD of 6 biological replicates. **j** Numbers of hCiPS cell colonies from different donors (*n* = 6). “6 + 4 + 2 + 6” represents a sequential duration (in days) for each stage. The short line indicates the mean of the indicated biological replicates. **k** Number of hCiPS cell colonies derived from small-amount blood samples (50 μL and 100 μL venous blood samples from two different donors). **l** Immunofluorescence staining of pluripotency markers in hCiPS cell colonies. Scale bar, 100 μm. **m** Schematic diagram of the experimental workflow for deriving primary hCiPS cell colonies from human peripheral blood cells under the serum-free conditions. For **b**, **e**, **g** and **i**, the *P* values were calculated using two-tailed unpaired *t*-tests.

To functionally replace FBS, we systematically tested and optimized the basal medium and supplements, and identified that a combination of B27 supplement and Knockout Serum Replacement (KSR) could partially substitute serum while maintaining morphological transformation and proliferative capacity of the PBMC-EPCs (Fig. 1a, b). Building upon this optimized platform, we further conducted a small molecule screen and found that PY-60, targeting annexin A2, could further enhance the emergence of epithelial-like cells during the initial reprogramming stage (Fig. 1a, b). Treatment of this serum-free condition downregulated blood cell identity-related markers while simultaneously accelerating the upregulation of key epithelial-related genes, facilitating the early phase change of cellular reprogramming (Fig. 1c; Supplementary Fig. S1a–c).

Upon transitioning to the stage 2 medium, we observed activation of LIN28A (Fig. 1d), a pluripotency marker that plays an essential role in the early stage of chemical reprogramming process. Following the treatment of late-stage reprogramming conditions, we successfully obtained hCiPS cell colonies that exhibited strong positive staining of the core pluripotency markers OCT4, SOX2, and NANOG (Fig. 1e, f; Supplementary Fig. S1d). Further refinement of small molecule concentrations revealed that precise dosing of 616452 (a TGF-β inhibitor) could facilitate the generation of hCiPS cells (Fig. 1g). We also tested the expansion duration of EPCs for reprogramming and revealed that an 8-day expansion is sufficient to generate hCiPS cells under the serum-free conditions (Supplementary Fig. S1e–h). Therefore, we successfully established a serum-free and clinical applicable chemical reprogramming system to generate hCiPS cells from adult blood cells.

To further validate the reproducibility of this serum-free chemical reprogramming system, we tested blood samples from 14 different donors. The results demonstrated that this serum-free system consistently generated hCiPS cells robustly across all tested donors, yielding > 100 hCiPS cell colonies in a single well of a 48-well plate (Fig. 1h; Supplementary Fig. S1l–m). Furthermore, to assess the reprogramming kinetics of the serum-free system, we tested treatment durations across multiple cell lines. The results showed that hCiPS cells could be induced with high efficiency within 22–24 days under this new reprogramming conditions (Fig. 1i; Supplementary Fig. S1j–k). Notably, hCiPS cells were successfully induced within as few as 18 days in this serum-free system (Fig. 1j).

We further tested whether our serum-free condition was suitable for small-amount blood samples. 50–100 μL venous blood was collected from different donors, treated with lysis buffer directly and cultured in EPC expansion medium. Then, these cells were reprogrammed under the serum-free conditions and after 24 days of treatment, we successfully generated hCiPS cells from these small-amount blood samples (Fig. 1k). Additionally, fingerstick blood provides a very easily accessible resource for cellular reprogramming, which would facilitate the development of large-scale hCiPS cell banks and applications of personalized therapies. We revealed that hCiPS cells could also be induced from fingerstick blood (Supplementary Fig. S1i). These results demonstrated the remarkable robustness and versatility of this serum-free chemical reprogramming approach, even successful with minimal-volume blood as starting material.

The hCiPS cells derived from this serum-free system were characterized for pluripotency and differentiation potentials. These established cell lines displayed morphological features including well-defined colony borders and prominent nucleoli (Supplementary Fig. S2a), which highly resemble human embryonic stem cells (hESCs). Karyotype analysis confirmed that these cells possessed normal chromosomal integrity (Supplementary Fig. S2b). Additionally, genetic fingerprinting through short tandem repeat profiling confirmed the origin of the hCiPS cells from their parental somatic counterparts (Supplementary Table S1). Immunofluorescence staining revealed co-expression of core pluripotency transcription factors, OCT4, SOX2, and NANOG, along with pluripotent surface markers (Fig. 1l; Supplementary Fig. S2c). Transcriptional analysis by RT-qPCR showed that the expression levels of pluripotency-associated genes in hCiPS cells were comparable to those in hESCs (Supplementary Fig. S2d). Global gene expression profiling through RNA sequencing further indicated that the blood-derived hCiPS cells highly resemble hESCs (Supplementary Fig. S2e, f). These results collectively demonstrated the successful acquisition and maintenance of pluripotent state in the hCiPS cells.

Next, we assessed the differentiation capacity of hCiPS cells. Firstly, embryoid body (EB)-mediated spontaneous differentiation was conducted, and immunofluorescence analysis clearly demonstrated the expression of ectodermal (SOX1/TUJ1), mesodermal (SOX9/GATA4), and endodermal (SOX17/FOXA2) lineage markers (Supplementary Fig. S2g). We also performed TaqMan hPSC scorecard assay and confirmed that serum-free blood-derived hCiPS cells were able to differentiate into three germ layers in vitro (Supplementary Fig. S2h). Furthermore, in vivo teratoma formation assays confirmed trilineage differentiation potential of hCiPS cells, with the generation of three germ layer-related tissues (Supplementary Fig. S2i). Taken together, these results fully characterized hCiPS cell lines and confirmed their abilities to differentiate into all three germ layers both in vitro and in vivo.

In this study, we established a robust, reproducible, and clinically compliant chemical reprogramming system for generating human pluripotent stem cells directly from peripheral blood (Fig. 1m). Notably, this reprogramming protocol enables the generation of hundreds of hCiPS cell colonies from a well of a 48-well plate (Fig. 1h), highlighting the high efficiency of the system. Crucially, the replacement of FBS with defined components eliminates the potential risks of pathogen contamination and immunogenic concerns, substantially enhancing the system’s suitability for further clinical translation. This study provides a simple and highly accessible strategy for generating large-scale, clinically-compliant hCiPS cells from diverse donor populations.

Importantly, this clinically-applicable reprogramming system would significantly advance the generation of human pluripotent stem cells, offering a next-generation platform for production of personalized stem cell lines. This convenient and serum-free method enables the generation of hCiPS cells without integrating any exogenous factors, allowing the cells to be readily used. In contrast, the traditional approaches, such as the episomal- and Sendai virus-based blood reprogramming systems, require prolonged passaging of the resulting iPS cell lines for eliminating the exogenous factors^9^. Additionally, the small molecule-based approach is more advanced for producing commercial kits that are cost-effective and only require step-by-step medium changes, without the need for complicated viral infection or plasmid/mRNA delivery^4,11,12^. Furthermore, small molecules are easy to synthesize, standardize, and manufacture^4,11^. Collectively, these advantages of chemical reprogramming of blood samples would greatly facilitate the production of hCiPS cells for broad clinical applications.

Additionally, for clinical translation of hCiPS cells, various functional cells have been demonstrated to be efficiently differentiated from hCiPS cells^8,13–15^. Notably, clinical study further showed that autologous transplantation of hCiPS cell-derived pancreatic cells enabled rapid restoration of insulin-independent glycemic control in type 1 diabetes patient^8^. These breakthroughs highlighted the immense potential of hCiPS technology for treating major diseases. Critically, the previously used hCiPS cells are derived from the patient’s adipose or skin tissues, requiring invasive procurement^7,8,12^. This limitation was overcome by blood reprogramming platform, which enables minimally invasive generation of hCiPS cell lines. With this robust, convenient and clinical-applicable conditions, blood chemical reprogramming strategy would significantly accelerate the clinical translation of hCiPS cells for personalized cell therapies.

## Supplementary information

Supplementary information

Supplementary Table S1

Supplementary Table S2

Supplementary Table S3

Supplementary Table S4

Supplementary Table S5

## Data Availability

The raw sequencing data can be accessed using the GSE accession number: bulk-RNA-seq (GSE305172).
