# Supplementary material for: Robust generation of clinically applicable human pluripotent stem cells from peripheral blood by chemical reprogramming
Source: Cell Discov. 2025 Dec 23;11:103. doi: 10.1038/s41421-025-00852-7 (PMC12727690; doi:10.1038/s41421-025-00852-7)
Supplement: Supplementary file 1 — Supplementary information [file 41421_2025_852_MOESM1_ESM.pdf]

Supplementary Information for

Title: Robust generation of clinically applicable human pluripotent stem cells from peripheral blood by chemical reprogramming

Author: Xiaodi Fu[1,6], Fangqi Peng[1,6], Ruyi Cai[2,6], Jingping Mao[1,6], Tianxing Liu[1,6], Yingshuai Dong[3,6], Ruoqi Cheng[1,4], Zhihan Yang[1], Guanxian Chen[1], Cheng Li[4], Rong Mu[2], Lin Cheng[1], Yanglu Wang[3,7], Jingyang Guan[3,7], Hongkui Deng[1,5,7]

Affiliations: [1]MOE Key Laboratory of Cell Proliferation and Differentiation, School of Life Sciences and MOE Engineering Research Center of Regenerative Medicine, School of Basic Medical Sciences, State Key Laboratory of Natural and Biomimetic Drugs, Peking University Health Science Center, Peking-Tsinghua Center for Life Sciences, Peking University, Beijing, China. [2]Department of Rheumatology and Immunology, Peking University Third Hospital, Beijing, China. [3]State Key Laboratory of Natural and Biomimetic Drugs, Department of Molecular and Cellular Pharmacology, School of Pharmaceutical Sciences, Peking University, Beijing, China. [4]School of Life Sciences, Center for Bioinformatics, Center for Statistical Science, Peking University, Beijing, China. [5]Changping Laboratory, Beijing, China. [6]These authors contributed equally: Xiaodi Fu, Fangqi Peng, Ruyi Cai, Jingping Mao, Tianxing Liu and Yingshuai Dong. [7]Corresponding author: Hongkui Deng; Jingyang Guan and Yanglu Wang. Email: hongkui_deng@pku.edu.cn; guanjingyang@hsc.pku.edu.cn; w_yanglu@126.com

This file includes:

Supplementary information, Figs. S1-S2

Legends of supplementary information, Tables S1-S5.

Materials and Methods

Supplementary information, Fig. S1

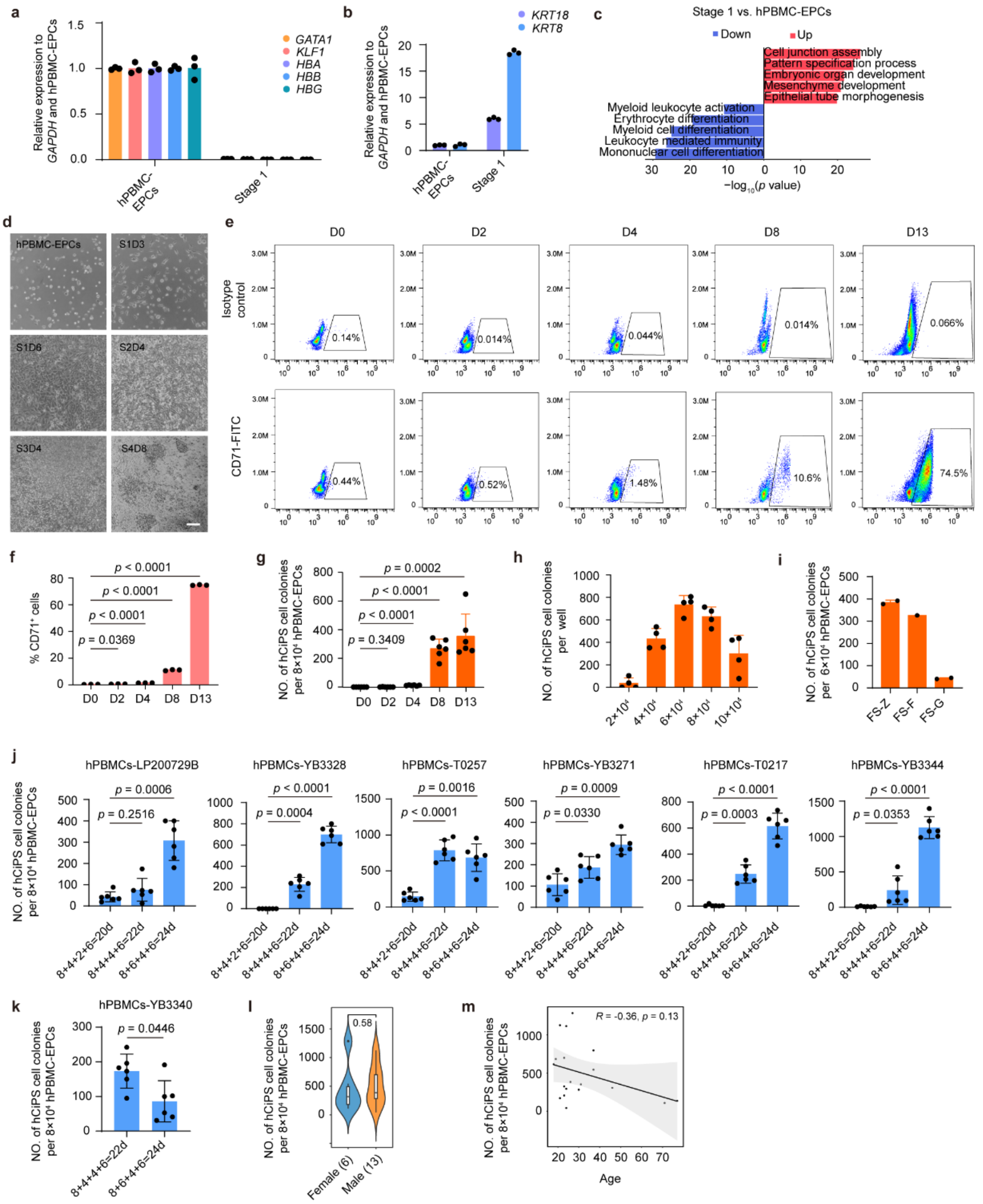

Supplementary information, Fig. S1. Establishment of a clinically-compliant reprogramming protocol for blood cells. a-b, RT-qPCR analysis of erythroid-related genes and epithelial related genes at the end of stage 1. Data are presented as mean (n = 3, technical replicates). **c**, GO analysis of differentially expressed genes between cells at stage 1 and hPBMC-EPCs. **d**, Morphologies of hPBMCs and cells at different time points (S1D3, S1D6, S2D4, S3D4, S4D6). Representative phase-contrast microscopy images were taken. Scale bar, 100 μm. **e**, FACS analysis of $CD71^{+}$ EPCs derived from hPBMCs cultured in the EPC expansion medium at the indicated

timepoints. Representative of three independent experiments. **f,** Percentage of $CD71^+$ cells over a 13-day time course in EPC culture condition. **g**, Number of hCiPS cell colonies generated from 8×10^4 cells starting at different culture timepoints of EPC expansion condition. Error bars indicate mean ± SD of 6 biological replicates. **h**, Numbers of hCiPS cell colonies with different EPCs seeding density (n=4). Error bars indicate mean ± SD of 4 biological replicates. **i**, Number of hCiPS cell colonies derived from cells isolated from fingerstick blood. **j**, Reprogramming kinetics across different donors in the serum-free protocol. Six independent hPBMC donors (YB3271, T0217, YB3344, T0257, LP200729B, and YB3328) were tested. Error bars indicate mean ± SD of 6 biological replicates. **k**, Reprogramming efficiency of hPBMC-YB3340 in the serum-free protocol (n=6). Error bars indicate mean ± SD of 6 biological replicates. **l**, Violin plot showed that the reprogramming efficiency of cells from donors of different genders. Significance was assessed by using a two-tailed Student's t test. **m**, Correlation analysis of the number of hCiPS cell colonies and donor age (R = -0.36, *p*=0.13). Points represent the replicates of the samples across the indicated ages. For s1f, s1g, s1j and s1k, the *p* values were calculated using two-tailed unpaired t tests.

Supplementary information, Fig. S2

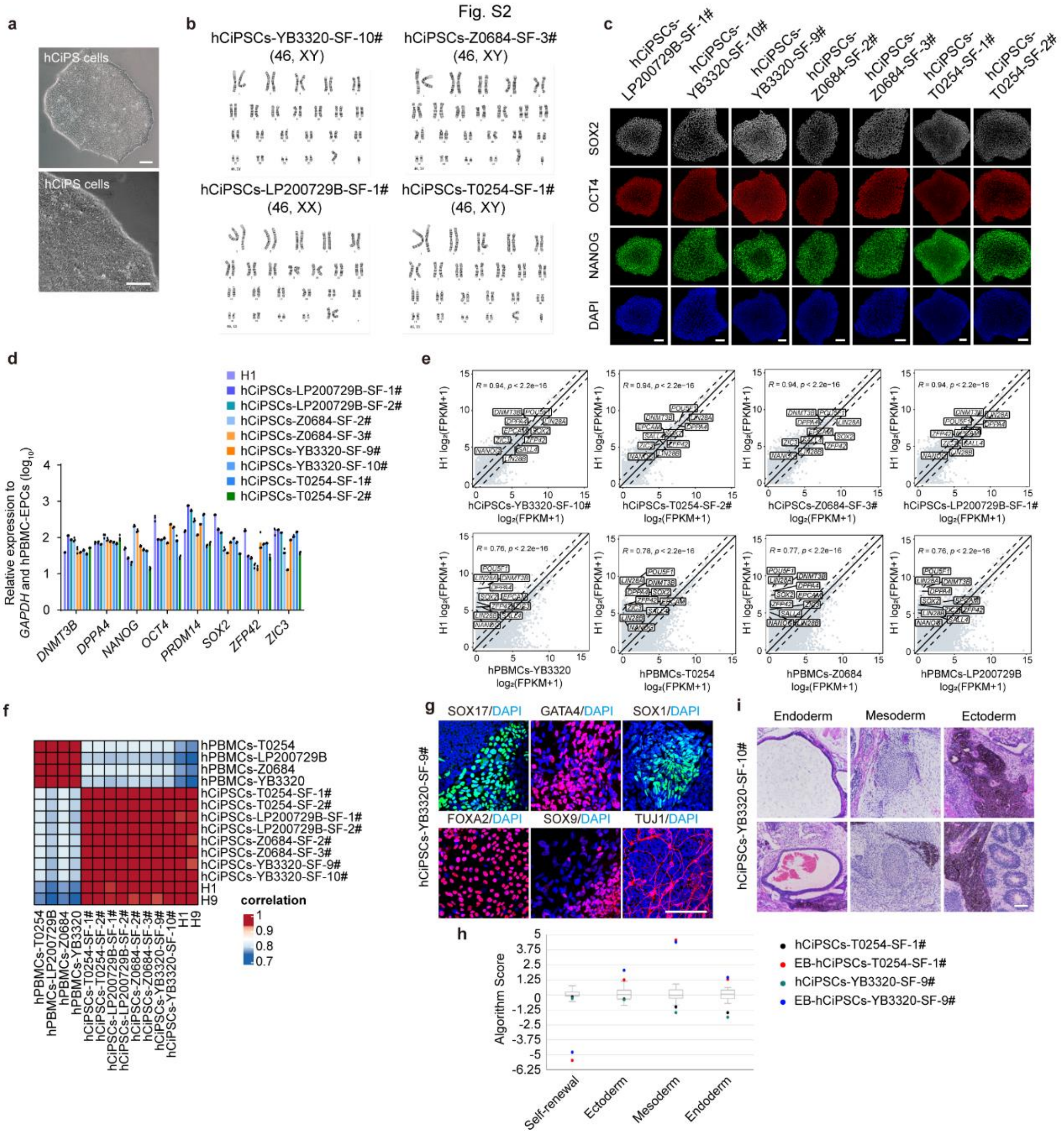

Supplementary information, Fig. S2. Characterization of hPBMC-derived hCiPS cells from the serum-free conditions. a, Representative phase-contrast microscopy images show typical hCiPS cell colony morphology. Scale bar, 100 μm. **b**, G-banded karyotypes of the indicated hCiPS cell lines confirm normal chromosomal content (46, XX or 46, XY). **c**, Immunofluorescent staining of pluripotency markers of hPBMC-derived hCiPS cell lines. Scale bar, 100 μm. **d**, RT-qPCR analysis of pluripotency-related genes in hCiPS cell lines derived from different donors. Data are presented as mean (n = 3, technical replicates). **e**, Scatter plots correlate gene expression of hCiPS cells vs hESCs (H1) and hPBMCs vs hESCs (H1). **f**, Correlation matrix heatmap

analyses based on the global transcriptional profile of hPBMCs, hESCs (H1 and H9) and hCiPS cell lines derived from different donors. **g**, Immunofluorescence of differentiation markers in hCiPSC-derived EBs. EBs were stained for germ-layer markers: ectoderm: SOX1/TUJ1, mesoderm: SOX9/GATA4, endoderm: SOX17 and FOXA2. DAPI (blue) stains nuclei. Scale bar, 100 μm. **h**, Data represent results from the TaqMan hPSC Scorecard assay, quantifying the pluripotency (self- renewal) and the trilineage differentiation capacity. **i,** Hematoxylin & eosin staining of the histological sections of teratomas formed by hCiPS cells display tissues from all three germ layers. Scale bar, 100 μm.

Supplementary information, Tables S1-S5.

Supplementary Table S1. Cell lines used in this study

Supplementary Table S2. Results of STR analysis

Supplementary Table S3. Small molecules, cytokines, other reagents and commercial kits

Supplementary Table S4. Antibody information

Supplementary Table S5. RT-qPCR primers

Materials and Methods

Animals

All animal experiments performed in this study were reviewed and approved by the Institutional Animal Care and Use Committee of Peking University, with strict adherence to the institution's Animal Protection Guidelines. Immunodeficient NPG mice were obtained from Beijing Vitalstar Biotechnology Company and housed in a controlled environment with a 12-hour light/12-hour dark cycle (6:00-18:00). The housing facility maintained a constant temperature of 22 ± 1 °C and a relative humidity of 40% and 60%, with free access to water and food.

Cell culture

hPBMCs and fingerstick blood cells were cultured in low-attachment cell culture plates (Corning) using EPC culture medium, consisting of StemSpan™ Serum-Free Expansion Medium II (SFEM II, STEMCELL) supplemented with 1% Penicillin-Streptomycin (Gibco), 100 ng/ml stem cell factor (SCF, Stemimmune LLC), 2U/ml erythropoietin (EPO, Stemimmune LLC), 40 ng/ml insulin-like growth factor 1 (IGF-1, Stemimmune LLC), 10 ng/ml interleukin-3 (IL-3, Stemimmune LLC), 250X Cholesterol Lipid Concentrate (Gibco) and 1 μM dexamethasone (MCE). Cultures were maintained at 37°C under 21% O_2 and 5% CO_2. hCiPS cells were cultured on Matrigel-coated culture plates in mTeSR™ Plus Medium. No evidence of cross-contamination or cell misidentification was observed, and no cell lines listed as misidentified by the International Cell Line Authentication Committee were utilized.

Isolation of hPBMC and fingerstick blood cells

Commercial hPBMCs were isolated from the venous blood of health donors and cultured in EPC medium. Human fingerstick blood cells were obtained with written informed consent from donors and approval by the Institute of Ethics Committee Review Board at Peking University (IRB 00001052-19070), in accordance with the Declaration of Helsinki. Approximately 50-100 μl of capillary blood was collected from individual donors in a sterile laboratory environment and treated with ACK lysing buffer to eliminate erythrocytes, typically yielding 4-8 x 10^5 cells. The resulting fingerstick blood cells were resuspended in 500 μl of EPC medium and seeded into

individual wells of 24-well low attachment cell culture plates (Corning). The detailed information of both hPBMCs and fingerstick blood cells was provided in Supplementary table S2.

Expansion of EPCs from hPBMCs and fingerstick blood cells

hPBMCs were cultured in EPC medium at a density of 5 x 10^6-1 x 10^7 per well in 6-well low-attachment cell culture plates (Corning). Fresh EPC medium was replenished every 48 hours without any medium aspirated. On day 7, cells were centrifuged, resuspended in fresh EPC medium, and replated at a density of 5 x 10^5 cells/mL (2 mL per well). From day 7 to 14, EPC medium was replenished using the same protocol as the first week (every 48 hours). Between days 12 and 14, cells were analyzed for the proportion of $CD71^+$ cells via flow cytometry (CytoFLEX, BECKMAN COULTER) and deemed suitable for chemical reprogramming. The EPC were immediately used for reprogramming without further passage.

Generation of hCiPS cells from hPBMCs and fingerstick blood cells

<u>Four sequential induction media were prepared for hCiPS cell generation:</u>

Stage 1 induction medium consisted of 80% KnockOut DMEM (Gibco) supplemented with 10% mTeSR^{TM} Plus Medium 5X supplement (STEMCELL, Component#100-0275), 2% B27 supplement (Gibco), 5% Knockout Serum Replacement (KSR, Gibco), 1% GlutaMAX, 1% non-essential amino acids (NEAA, Gibco), 1% Penicillin-Streptomycin (Gibco), 50 μg/ml ascorbic acid 2-phosphate (Vc2p), 1 ng/ml interleukin-1β (IL-1β, Peprotech), and the following small molecules: CHIR-99021 (5 μM), 616452 (10 μM), TTNPB (1 μM), Forskolin (50 μM), EPZ-5676 (4 μM), VTP50469 (1 μM), SETD2-IN-1(0.4 μM), Pro-CBP/P300-d (1 μM), AU-15330 (1 μM), EPZ-6438 (0.5 μM), BI-7273 (1 μM), NIBR-LTSi (2 μM), CX-4945 (2 μM) and PY-60 (5 μM).

Stage 2 induction medium was formulated using 90% KnockOut^{TM} DMEM supplemented with 2% B27 supplement, 5% KSR, 1% GlutaMAX, 1% NEAA, 1% Penicillin-Streptomycin, 50 μg/ml Vc2p, 40 ng/ml bone morphogenetic protein 4 (BMP4), and the small molecules: CHIR-99021 (5 μM), 616452 (10 μM), TTNPB (2 μM), SAG (0.5 μM), EPZ-5676 (2 μM), VTP50469 (0.5 μM), and AKT Kinase Inhibitor (1 μM), JNK-IN-8 (0.2 μM), SETD2-IN-1(0.2 μM), WM-8014 (1 μM), A-485 (0.5 μM), SGC-SMARCA-BRDVIII (2 μM) and CCS1477 (0.05 μM).

Stage 3 induction medium contained 95% KnockOut™ DMEM supplemented with 2% B27 supplement, 1% GlutaMAX, 1% NEAA, 1% Penicillin-Streptomycin, 50 μg/ml Vc2p, 200 ng/ml basic fibroblast growth factor (bFGF), and the small molecules: CHIR-99021 (5 μM), 616452 (10 μM), TTNPB (2 μM), SAG (0.5 μM), Y-27632 (10 μM), JNK-IN-8 (0.5 μM), EPZ-5676 (2 μM), DZNep (0.2 uM), Ruxolitinib (1 μM), BIRB 796 (2 μM), SGC-CBP30 (2 μM), VTP50469 (0.5 μM), and 5-Iodotubercidin (0.5 μM), AKT Kinase Inhibitor (0.5 μM), CX-4945 (0.5 μM), Retinoic acid (5 μM), GSK-3685032 (0.05 μM) and CCS1477 (0.2 μM).

Stage 4 induction medium contained 90% KnockOut™ DMEM supplemented with 2% B27 supplement, 5% KSR, 1% GlutaMAX, 1% NEAA, 1% Penicillin-Streptomycin, 50 μg/ml Vc2p, 20 ng/ml recombinant human heregulin β-1 (HRG) and the small molecules: CHIR-99021 (1 μM), Y-27632 (10 μM), PD0325901 (1 μM), SB-590885 (0.5 μM); this formulation was designated as Stage 4 Basal Medium.

For the initial 3 days of stage 4, valproic acid sodium salt (VPA; 200 μM), PY-60 (20 μM), tranylcypromine (10 μM), DZNep (0.2 μM), EPZ-5676 (2 μM) and SGC-SMARCA-BRDVIII (2 μM) were added to the Stage 4 Basal Medium. For the subsequent 3 days, only Stage 4 Basal Medium was used. To facilitate growth of primary hCiPS cell colonies, Stage 4 Basal Medium could be extended for additional 2-4 days.

The detail information of small molecules and cytokines is provided in the Supplementary table S3.

<u>Induction process of hCiPS cells derived from hPBMCs and fingerstick blood cells</u>

Hypoxic conditions (5% O_2) were maintained during Stages 1 and 2; following Stage 2 cultures were transitioned to 21% O_2.

1. Stage 1: hPBMCs (pre-expanded in EPC medium for 12-14 days) were seeded in Stage 1 medium at a density of 8 x 10^4 cells/well in fibronectin (Acro) -coated 48-well plates or 1.6 × 10^5 cells/well in 24-well plates. Due to limited cell numbers, fingerstick blood cells were seeded into 1–2 wells of 48-well plates. Cells adhered to the plate surface within 1 day, with proliferative clusters emerging by days 3–4. Clusters expanded in size and number, reaching 80–90% confluence over 6–8 days (avoiding

over-confluence). Medium was not refreshed during Stage 1, and cultures were transitioned to Stage 2 medium upon reaching the target confluence.

2. Stage 2: Cells adopted an epithelial-like morphology and reached 100% confluence over 4–6 days. Medium was refreshed every 4 days, after which cultures were switched to Stage 3 medium.

3. Stage 3: Multi-layered cell colonies formed and expanded. After 4 days in Stage 3 medium, cultures were transitioned to Stage 4 medium.

4. Stage 4: For the first 3 days, Stage 4 Basal Medium was supplemented with VPA (200 μM), PY-60 (20 μM), tranylcypromine (10 μM), DZNep (0.2 μM), EPZ-5676 (2 μM), and SGC-SMARCA-BRDVIII (2 μM). For the next 3 days, these supplements were omitted, and only Stage 4 Basal Medium was used. Immunofluorescent staining for OCT4 was performed at the end of Stage 4, with OCT4-positive colonies identified as primary CiPS cells.

5. Stage 4 could be extended for additional 2-4 days to promote further growth of primary hCiPS cell colonies.

Derivation and culture of hCiPS cell lines

After 6-8 days of Stage 4 induction, primary hCiPS cell colonies were dissociated using Accutase (Millipore), centrifuged at 300 x g for 3 minutes, and replated at a 1:3 to 1:12 ratio onto Laminin-521 (STEMCELL)-coated plates in Derivation Medium. This medium consisted of Knockout^{TM} DMEM supplemented with 2% B27 supplement, 5% KSR, 1% GlutaMAX, 1% NEAA, 1% Penicillin-Streptomycin, 50 μg/ml Vc2p, and the small molecules: CHIR-99021 (1 μM), PD0325901 (0.5 μM), Y-27632 (10 μM), HRG (20 ng/mL), and bFGF (80 ng/mL). Cultures were maintained at 37°C in 21% O_2 and 5% CO_2, with medium refreshed every four days. After 10-12 days, compact hCiPS cell colonies were mechanically dissociated into small clumps and transferred to Matrigel-coated plates. Then, these cells were cultured in mTeSR™ Plus Medium supplemented with Y-27632 (10 μM) for 24 hours, after which medium was replaced by fresh mTeSR™ Plus Medium without Y-27632.

Flow cytometry

Cells were adjusted to a density of $1x10^7$ cells/mL in pre-chilled FACS Buffer (PBS containing 2% fetal bovine serum (FBS, Gibco) and 3% Penicillin-Streptomycin**).** For surface marker detection, antibodies were diluted in FACS buffer and incubated with

cells for 30 min on ice in the dark. Cells were then filtered through a 40 μm cell strainer prior to analysis using a CytoFLEX flow cytometer (Beckman) system.

Immunofluorescence

Cells were fixed with 4% paraformaldehyde for 30 minutes at room temperature, then blocked for 1 hour at 37°C in PBS supplemented with 0.1% Triton X-100 (Sigma-Aldrich) and 2% normal donkey serum (Meilunbio). Primary antibodies, diluted to optimal concentrations, were incubated with cells in the same blocking buffer overnight at 4°C. After washing, cells were incubated with secondary antibodies in PBS containing 2% normal donkey serum for 2 hours at room temperature. Nuclear staining was achieved using DAPI solution (Roche). The detailed information of the antibodies was provided in the Supplementary Table S4.

Reverse transcription (RT)-quantitative PCR (qPCR)

Total RNA was extracted using the Magicpure® 32 Tissue/Cell Total RNA Isolation Kit (TransGen) on an automated nucleic acid extraction system (Hollycon, AE2120-32). Complementary DNA (cDNA) was synthesized from 1 μg of total RNA using HiScript III All-in-One RT SuperMix (Vazyme). Quantitative PCR was performed with PerfectStart® Universal Green qPCR SuperMix (TransGen) on a QuantStudio™ 5 Real-Time PCR Systems (Thermo Fisher Scientific). Target gene expression levels were normalized to *GAPDH* as the internal reference, and data were analyzed using the ΔΔCt method. Primer sequences are listed in the Supplementary Table S5.

RNA sequencing (RNA-seq)

Total RNA was extracted using the Magicpure® 32 Tissue/Cell Total RNA Isolation Kit (TransGen) on an automated nucleic acid extraction system (Hollycon, AE2120-32). RNA-seq libraries were prepared using the NEBNext Ultra RNA Library Prep Kit for Illumina (New England BioLabs, catalog no. E7775), which involves RNA fragmentation and random priming. Libraries were sequenced as 2 x 150 base pairs paired-end reads on an Illumina NovaSeq 6000 platform.

Bulk RNA-seq data analysis

Raw sequencing data quality was assessed using FastQC. Adapters and low-quality reads were trimmed with Trimmomatic[1] and cleaned reads were aligned to the human

reference genome (hg38) using STAR[2]. Gene expression counts were generated with featureCounts[3] and expression levels were normalized as fragments per kilobase million (FPKM). Differential gene expression analysis was performed using the R package DESeq2[4]. Differentially expressed genes (DEGs) were defined as those with a $\log_2$ fold change >1 and a p value <0.05, with thresholds adjusted for sample groups with more pronounced or subtle differences as needed. Heatmaps were generated using the R package ComplexHeatmap with scaled FPKM values.
GO enrichment analysis of DEGs or gene sets was performed using the enrichGO function in the R package. ClusterProfiler[5] with significant terms defined as those with p value <0.05. Spearman correlation coefficients were calculated, and results were visualized using scatter plots (ggpubr package) and heatmaps (corrplot package).

Karyotype and short tandem repeat (STR) analysis

For karyotyping, high-resolution G-banding (400G-500G) was conducted by Beijing Jiaen Hospital using standard protocols. At least 20 metaphase cells were examined per sample to assess chromosome number and structure, with data analyzed using CytoVision (Leica).
STR analysis was performed by Beijing Microread Genetics: genomic DNA was extracted using a Microread Genomic DNA kit, amplified via PCR with an STR Multi Amplification kit (Microreader 21 ID System), and analyzed on an ABI 3730xl DNA Analyzer (Applied Biosystems). Data were processed using GeneMapperID-X software, with 21 loci examined per sample; no cross-contamination with other cell lines was detected. Results were provided in Supplementary Table S1.

Teratoma formation

For teratoma formation, approximately 2 x 10^6 hCiPS cells were harvested with ReLeSR, resuspended in Matrigel, and injected into the renal subcapsular space of 2- to 3-month-old immunodeficient NPG mice (Vitalstar Biotechnology). Teratomas typically developed within 4-6 weeks post-injection. Samples were embedded in paraffin, sectioned, and stained with hematoxylin and eosin. All animal experiments were approved by the Institutional Animal Care and Use Committee of Peking University and conducted in accordance with the Animal Protection Guidelines of Peking University.

Embryoid bodies (EBs) formation

hCiPS cells were enzymatically dissociated into small clumps using ReLeSR™ and cultured as three-dimensional spheroids on ultra-low attachment culture plates for 24 hours in mTeSR™ Plus medium supplemented with Y-27632 (10 μM). Directed differentiation was initiated by transferring spheroids to high-glucose Dulbecco's Modified Eagle Medium (DMEM) supplemented with 20% FBS, with culture maintained under standard conditions for 16 days. Resultant EBs were plated onto Matrigel-coated substrates and cultured in the same differentiation medium for 14-21 days. Terminal differentiation samples were analyzed by immunofluorescence using a confocal microscope (STELLARIS 8, Leica) with lineage-specific markers. Alternatively, pluripotency and differentiation efficiency were quantified using the TaqMan™ hPSC Scorecard™ Panel (Thermo Fisher Scientific), a validated platform for profiling human pluripotent stem cell characterization. Gene expression data were analyzed using the web-based hPSC Scorecard™ Analysis Software to assess pluripotency and differentiation potential.

Statistical analysis

The number of biological replicates and the methods used for the statistical tests are described in the figure legends. GraphPad Prism 8 was used for statistical analysis. *P* values were calculated using two-tailed unpaired t tests unless otherwise stated and are shown in the related figures. A *P* value <0.05 was considered to indicate statistical significance.

Reference:

1 Bolger, A. M., Lohse, M. & Usadel, B. Trimmomatic: a flexible trimmer for Illumina sequence data. *Bioinformatics* **30**, 2114-2120 (2014). https://doi.org:10.1093/bioinformatics/btu170

2 Dobin, A. *et al.* STAR: ultrafast universal RNA-seq aligner. *Bioinformatics* **29**, 15-21 (2013). https://doi.org:10.1093/bioinformatics/bts635

3 Liao, Y., Smyth, G. K. & Shi, W. featureCounts: an efficient general purpose program for assigning sequence reads to genomic features. *Bioinformatics* **30**, 923-930 (2014). https://doi.org:10.1093/bioinformatics/btt656

4 Love, M. I., Huber, W. & Anders, S. Moderated estimation of fold change and dispersion for RNA-seq data with DESeq2. *Genome Biol* **15** (2014). https://doi.org:ARTN 55010.1186/s13059-014-0550-8

5 Yu, G. C., Wang, L. G., Han, Y. Y. & He, Q. Y. clusterProfiler: an R Package for Comparing

Biological Themes Among Gene Clusters. *Omics* **16**, 284-287 (2012). https://doi.org:10.1089/omi.2011.0118
